# Postoperative adjuvant radiotherapy for patients with upper tract urothelial carcinoma (UTUC) who underwent kidney-sparing surgery (KSS): a single-center study

**DOI:** 10.1186/s13014-023-02303-7

**Published:** 2023-07-18

**Authors:** Hui Guan, Guangyu Wang, Weiping Wang, Yuncan Zhou, Zhikai Liu, Xiaorong Hou, Junfang Yan, Shuai Sun, Ke Hu, Jing Zhao

**Affiliations:** 1 Department of Radiation Oncology, Peking Union Medical College Hospital, Chinese Academy of Medical Sciences and Peking Union Medical College, Beijing, People’s Republic of China; 2 Department of Oncology, Beijing Shijitan Hospital, Capital Medical University, Beijing, People’s Republic of China

**Keywords:** Kidney-sparing surgery, Upper tract urothelial carcinoma, Adjuvant radiotherapy

## Abstract

**Objective:**

The purpose of this study was to evaluate the efficacy of postoperative adjuvant radiotherapy for patients with upper tract urothelial carcinoma (UTUC) who underwent kidney-sparing surgery (KSS).

**Methods:**

We retrospectively reviewed the clinical records of 31 patients with primary UTUC who underwent kidney-sparing surgery (KSS) and who were treated with adjuvant radiotherapy at our center between October 1998 and May 2017. Statistical analyses were performed with SPSS 23.0. The primary endpoints of this study included overall survival (OS) and local recurrence-free survival (LRFS); the secondary endpoints were disease-free survival (DFS) and treatment-related toxicity.

**Results:**

The median follow-up was 58.4 months (range, 12.7-185.3 months), and the median local recurrence time was 59.0 months (range, 7.0-185 months). All of the patients completed radiotherapy on schedule, and no grade 3–4 late-stage reaction was observed. The estimated 5-year and 10-year OS, DFS and LRFS rates of the patients were 64.0%, 61.1%, 69.6% and 48.0%, 40.9%, 64.6%, respectively. Univariate analysis showed that age (χ2 = 4.224, *P* = 0.040), R0 resection (χ2 = 3.949, *P* = 0.047), and early stage (I + II) (χ2 = 6.515, *P* = 0.011) were associated with good OS; DFS benefit in early stage patients (χ2 = 6.151, *P* = 0.013) and age<70 years old (χ2 = 5.091, *P* = 0.024). Patients with distal ureteral segments had better LRFS than patients with proximal ureteral cancer (χ2 = 5.248, *P* = 0.022). However, multivariate analysis showed that age was the only factor of OS (χ2 = 4.099, *P* = 0.043).

**Conclusion:**

Adjuvant radiotherapy is safe and tolerated, and LRFS was superior in middle and distal ureteral cancer than in proximal ureteral cancer.

## Introduction

Upper tract urothelial carcinoma (UTUC) is an uncommon cancer accounting for approximately 5–10% of all urothelial carcinomas [1]. Nearly 60% of UTUC patients have invasive disease, and approximately 25% of patients have regional metastasis at the time of diagnosis. Radical nephroureterectomy (RNU) with bladder-cuff removal is the gold standard treatment for high-risk UTUC with a good outcome for early-stage patients [2]. However, postoperative adjuvant therapies are limited due to the significant loss of renal function after removal of a renal unit during RNU, and administration of adjuvant chemotherapy is challenging. It has been reported that the 5-year cancer-specific survival rate for patients with pT2/pT3 tumors is less than 50%, and it is less than 10% for patients with T4 or N + disease [3]. Recently, for patients with low-risk UTUC, including the following factors: unifocal disease, tumor size < 2 cm, low-grade cytology, low-grade ureteroscopic biopsy and no invasion on CT urography (CTU), there has been an increasing tendency toward kidney-sparing surgery (KSS), including segmental ureterectomy (SU) [4]. A meta-analysis showed that there was no difference in HR for cancer-specific survival (CSS) or overall survival (OS) between RNU and SU [5], but the risk of local and bladder recurrence remains relatively high. In consideration of a 30–50% local recurrence rate even when postoperative chemotherapy is conducted [6–8], it is necessary to initiate new strategies to improve efficacy.

Postoperative radiotherapy can significantly improve the local control rate in many carcinomas [9]. However, the efficacy of postoperative radiotherapy in UTUC is unclear due to the rarity of the disease. Recent studies have shown that adjuvant radiotherapy can decrease the 3-year local recurrence rate (LRR) from 39 to 11% and improve overall survival [10, 11]. Liu et al. explored the potential safety and effectiveness of radiotherapy as a curative treatment for UTUC patients who cannot undergo surgery for several reasons, such as unresectable disease, old age, and multiple comorbidities [12]. Because of the high risk of local and bladder recurrence, vigilant radiographic and endoscopic surveillance of the bladder and the ipsilateral upper tract are required after KSS [4]. However, the efficacy and safety of postoperative adjuvant radiotherapy in patients with KSS have not been reported. Meanwhile, previous research results in our center and with a small sample study showed that there was no significant difference in OS between patients treated with postoperative radiotherapy in the non-RNU group and RNU group, so we think that patients with KSS may benefit from postoperative adjuvant radiotherapy [13]. In this article, we further investigated the efficacy of postoperative adjuvant radiotherapy for patients with UTUC who suffered from KSS.

## Methods

We retrospectively reviewed the clinical records of patients with primary UTUC who suffered from KSS treated with adjuvant radiotherapy at our center between October 1998 and May 2017. The eligibility criteria were as follows: KSS was conducted and postoperative histologically confirmed UTUC; no distant metastasis; and complete follow-up data were available. Pretreatment evaluation included complete history and physical examination, blood count, renal and liver function, chest and abdomen CT, pelvic magnetic resonance imaging (MRI) and transrectal ultrasound.

### Treatment

All patients received a CT simulation (16-slice Philips Brilliance BigBore CT, Netherland) in the supine position with oral and intravenous contrast agents. The gross tumor volume (GTV) and clinical target volume (CTV) were contoured on axial CT slices. The GTV included the primary tumor (GTV-T), which indicated pathological R1 (microscopic positive margins), and the involved lymph nodes (GTV-N). CTV covered GTV-T, GTV-N (if any), and pelvic lymph node regions (including common iliac, internal iliac, external iliac and obturator lymph nodes on the same side of the lesion). When the tumor was located in the upper ureter, the CTV covered the primary tumor, and when the tumor was located in the middle and distal ureter, the field of CTV was extended, including the adjacent ureteral passage region and the bladder or the lymphatic drainage region. Different radiotherapy regimens were given according to the patients’ condition. The planning target volume (PTV) consisted of the CTV with a 5 mm margin expansion. For R1 or lymph node metastasis, a boost of 4 to 20 Gy at 2 Gy per fraction was considered.

### Follow-up and evaluation of toxicities

The patients were followed up every 3 months within 2 years after radiotherapy, once every 6 months after 2 years, and once every 5 years after 5 years. The review items included computed tomography (CT), B-scan ultrasonography, and magnetic resonance urography (MRU). Other examinations, including routine blood examination, voided urine cytology, chest radiography, and cystoscopic examination, were also performed during follow-up. Patients with acute and chronic radiotherapy toxicity were evaluated according to the National Cancer Institute Common Toxicity Criteria for Adverse Events (CTCAE), version 5.0.

### Statistics

The primary endpoints of this study included overall survival (OS), local recurrence-free survival (LRFS), disease-free survival (DFS), and treatment-related toxicity. OS was defined as the time to death or the last follow-up from the beginning of treatment; DFS was defined as the time to recurrence, metastasis or last follow-up from the beginning of treatment; LRFS was defined as the time to recurrence of the primary tumor (including bladder) or the last follow-up from the beginning of treatment. Survival curves were calculated by the Kaplan‒Meier method and compared using the log-rank test. The Cox proportional hazards model was used to determine the independent factors affecting endpoints based on the variables selected by the univariate analysis. The chi-square test was used for comparisons between groups. Statistical analyses were performed with SPSS 23.0 for Windows (SPSS, Inc., Chicago, IL). P values < 0.05 were considered statistically significant.

## Results

The last follow-up was December 31, 2020. Two patients lost to follow-up within one year after radiotherapy were excluded, and a total of 31 patients were enrolled in this study. The median age was 65 years old (range 39–81 years). There were 11 patients ≥ 70 years old, including 6 patients over 75 years old. Two patients had a previous history of bladder cancer of more than 5 years. One patient had a history of colon cancer surgery, and 6 patients had pathological R1. According to the AJCC 8th edition [14], there were 20 (64.5%) and 11 (35.5%) patients with stage I-II and stage III-IV disease, respectively. The major histological types were well-differentiated transitional epithelial cell carcinoma. There were two cases of rare pathological types, including poorly differentiated adenocarcinoma and poorly differentiated squamous cell carcinoma. Most tumors (83.9%) were located in the middle and distal ureter. Renal pelvis involvement occurred in 3 patients, bladder involvement in 4 patients and multifocal disease involvement in 3 patients. Detailed clinical data of these patients are detailed in Table 1.

**Table 1:** Demographic data

Characteristics		n (%)
Age (year)	Median 65 (range, 39–81)	
Gender		
	Male	17(54.8)
	Female	14(45.2)
Complicated with chronic diseases		15(48.4)
Pathological differentiation		
	well differentiation	28(90.3)
	poor differentiation	3(9.7)
Tumor position		
	proximal segment	5(16.1)
	middle and distal segment	26(83.9)
Surgical margrin status		
	R0	25(80.6)
	R1	6(19.4)
pT classification		
	T1	8(25.8)
	T2	12(38.7)
	T3	9(29.0)
	T4	2(6.5)
pTNM staging		
	I	8(25.8)
	II	12(38.7)
	III	9(29.0)
	IV	2(6.5)
Risk stratification		
	Low-risk	20(64.5)
	High-risk	11(35.5)
Postoperative chemotherapy		
	Yes	4(12.9)
	No	27(87.1)
Post-radiotherapy treatment		
	Yes	5(16.1)
	No	26(83.9)
Duration of radiotherapy	Median 36 days(range 32-62 days)	

### Treatment and acute toxicities

A total of 4 patients received postoperative chemotherapy due to high-risk pollution. The median time from the operation to the onset of radiation therapy was 41 days (13–229 days), with a median treatment time of 36 days (32–62 days). Three-dimensional conformal radiotherapy (3D-CRT) was conducted in 11 patients, and intensity-modulated radiotherapy (IMRT) was conducted in 20 patients. The median radiotherapy dose was 50 Gy (range 30-61.52 Gy, 1.8 ~ 2.0 Gy/fraction). For 21 patients with distal ureter tumors, 13 of the patients (52%) had the field of CTV that covered the middle ureter and bladder. During the treatment period, there were 8 cases of grade 1–2 acute hematological toxicity, 6 cases of grade 1–2 gastrointestinal toxicity, 10 cases of grade 1–2 urinary system toxicity, and 1 case of concurrent chemotherapy with grade 3 myelosuppression. All of the patients completed radiotherapy on schedule, and no grade 3–4 late-stage reaction was observed.

### Pattern of failure and survival

**Fig. 1:**
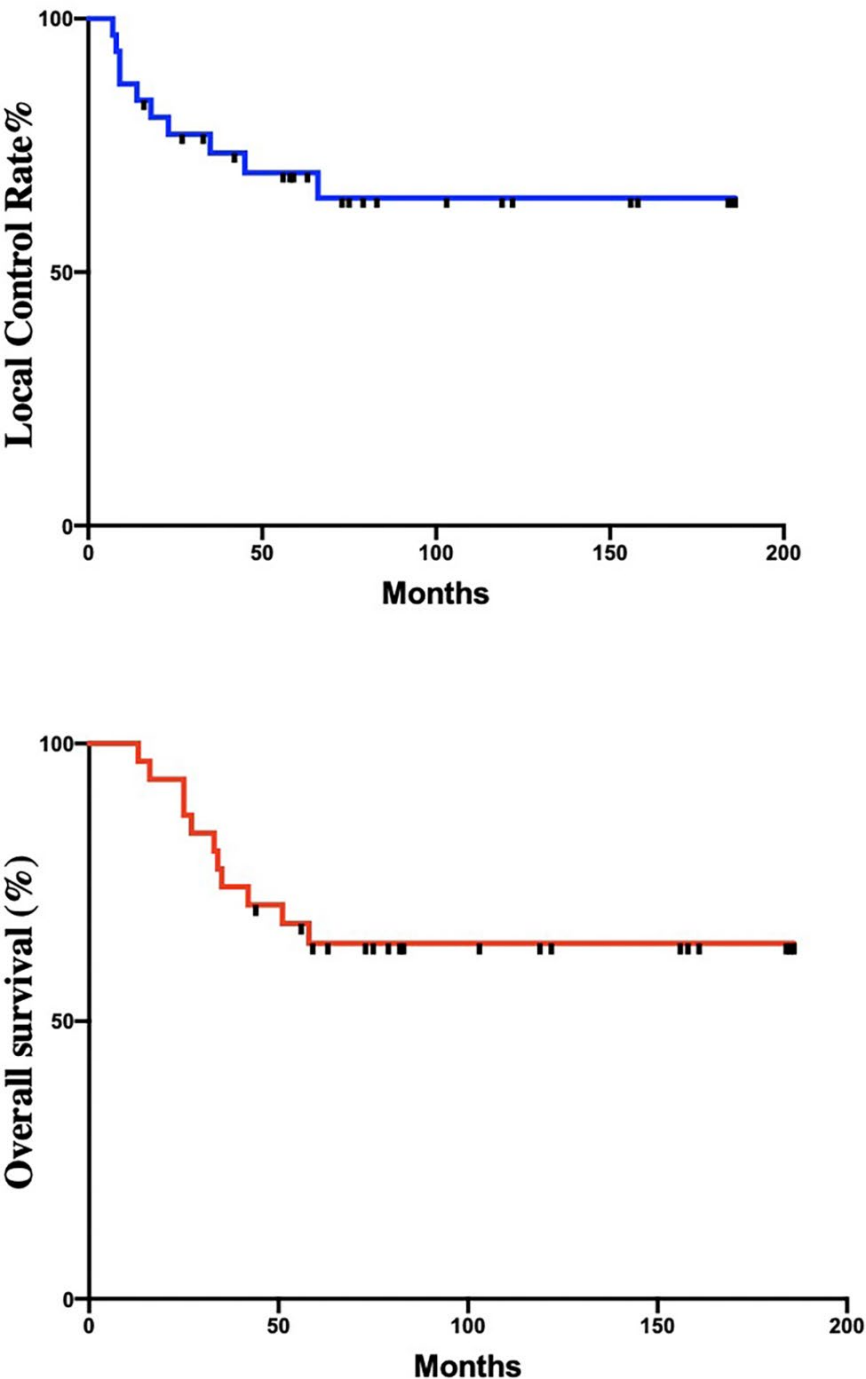
Overall survival curve and local control rate of patients

The median follow-up was 58.4 months (range, 12.7-185.3 months), and the median local recurrence time was 59.0 months (range, 7.0-185 months). A second malignant tumor occurred in 1 patient with adenolymphoma of the parotid gland. Twelve patients (38.7%) died due to disease progression. Distant metastasis occurred in 17 patients (54.8%), and local recurrence occurred in 10 patients (32.3%), including 4 patients (12.9%) with bladder recurrence. The estimated 5-year and 10-year OS, DFS and LRFC rates of patients were 64.0%, 61.1%, and 69.6% and 48.0%, 40.9%, and 64.6%, respectively. The OS and LC rate curves are shown in Fig. 1. Univariate analysis showed that age (χ2 = 4.224, *P* = 0.040), R0 resection (χ2 = 3.949, *P* = 0.047), and early stage (I + II) (χ2 = 6.515, *P* = 0.011) were associated with good OS; DFS benefit in early stage patients (χ2 = 6.151, *P* = 0.013) and age<70 years old (χ2 = 5.091, *P* = 0.024). Patients with distal ureteral segments had a better LRFC than patients with proximal ureteral cancer (χ2 = 5.248, *P* = 0.022). The analysis of prognostic factors is shown in Table 2. However, multivariate analysis showed that age was the only factor of OS (χ2 = 4.099, *P* = 0.043).

**Table 2:** Analysis of prognostic factors

		OS			DFS			LRFC	
		χ2	*p*		χ2	*p*		χ2	*p*
Age		4.224	0.040		5.091	0.024		2.675	0.102
	<70ys								
	≥ 70ys								
R0 resection		3.949	0.047		1.844	0.175		0.193	0.660
	Yes								
	No								
Pathological differentiation		0.036	0.850		0.083	0.774		0.015	0.902
	well differentiation								
	poor differentiation								
Tumor location		1.213	0.271		2.187	0.139		5.248	0.022
	Proximal								
	middle and distal								
Stage TNM		6.515	0.011		6.151	0.013		0.003	0.955
	I + II								
	III + IV								
Population characteristics		2.882	0.090		2.448	0.118		0.003	0.955
	low-risk								
	high-risk								
Radiotherapy techniques		1.041	0.308		0.290	0.590		0.148	0.700
	3-DCRT								
	IMRT								
Duration of radiotherapy		2.454	0.117		0.642	0.423		0.164	0.686
	<35days								
	≥ 35days								

## Discussion

UTUC is a rare urinary system tumor, accounting for only 5% of all urinary system tumors in the Western population. The incidence in the Asian population has increased slightly as a result of taking traditional Chinese medicines containing aristolochic acid. UTUC accounts for 20–25% of all urinary system tumors. The prognosis of primary ureteral carcinoma is poor. This may be due to the thin wall of the ureter, which allows the tumor to pass through the basal layer easily, and the rich lymph around the ureter is prone to local invasion and metastasis. Good results can be obtained by a simple operation in patients with early ureteral carcinoma. Hall et al. reported that the 5-year disease-free survival rates of patients with pTa, pT1, pT2, pT3 and pT4 were 100%, 91.7%, 72.6%, 40.5% and 0%, respectively. However, a number of studies have shown that for patients with tumors above stage III, the OS rate of more than 5 years ranges from 12 to 64% [2, 11, 15, 16].

In UTUC, due to the significant loss of renal function after removal of a rental unit during RNU, administration of adjuvant chemotherapy is challenging [17, 18]. Recent large-scale evidence from the National Cancer Database (NCDB) showed that patients with locally advanced or node-positive disease could benefit from adjuvant treatment (HR 0.77). However, no studies have shown that adjuvant chemotherapy had a survival benefit for patients with KSS [19]. In our study, only 4 (11.1%) patients were treated with postoperative chemotherapy, and there was no significant difference in OS and DFS due to the small sample size.

The effectiveness of postoperative adjuvant radiotherapy for UTUC has been controversial. Chen et al. [11] and Fan K et al. [20] showed that postoperative radiotherapy could improve the OS of patients with T3-4 tumors. Li HZ et al.’s [21] studies showed that adjuvant radiotherapy significantly improved 5-year RFS compared with partial ureterectomy alone, and partial ureterectomy combined with adjuvant radiotherapy did not significantly improve 5-year RFS compared with radical nephroureterectomy. Jwa E et al. [10] showed that adjuvant radiotherapy could improve the local control rate and could reduce the bladder recurrence rate but did not improve OS or PFS. Chang YH et al.’s [22] study yielded similar results. However, a KSS approach is advocated for low-risk tumors or for high-risk tumors when there is an imperative indication (renal insufficiency or solitary functioning kidney), and whether postoperative adjuvant radiotherapy is beneficial in patients with KSS has not been studied. In our respective study, the estimated 5-year OS, DFS and LC rates of patients were 64.0%, 61.1%, and 69.6%, respectively. The effective rates were higher than those of patients who underwent surgery alone reported in the literature [23, 24] and similar to those of postoperative radiotherapy groups [11, 25]. Bladder recurrence is common postoperatively in patients with UTUC and a risk factor for a poor prognosis [26]. The bladder recurrence rate can reach 27 to 47% [27, 28] after surgery, as described in the literature. In our study, the bladder recurrence rate was 12.9%, much lower than that in previous reports.

At present, there is no consensus on the radiation range of postoperative radiotherapy for UTUC. In most centers, CTV covers only the tumor bed area, the regional lymph node drainage area, and the retroperitoneal lymph node drainage areas with definite or suspected lymph node metastasis [20, 22, 25, 29]. However, in 2011, Chen’s study clearly defined the irradiation range, which included the renal pelvis, the full length of the ureter, the whole bladder and the retroperitoneal lymph node drainage area, in 53 patients – the most extensive group examined among all known studies. Chen et al. also mentioned that radiotherapy reduces the incidence of bladder recurrence (38.7–13.2%, p < 0.001) and recommended that the target area of radiotherapy include the whole bladder [11]. Belhadj Y found that bladder recurrence could occur anywhere in postoperative patients with UTUC [30], which also supports whole bladder irradiation. However, the rate of acute bladder toxicity (grades 1 and 2) was 58.2%, and the rate of grade 3 or above toxicity was 3%. Meanwhile, the literature showed that patients with lower ureteral tumors had a higher prevalence of deaths (HR = 2.227) than patients with upper ureteral tumors [31]. Therefore, in our study, the target volume covering the bladder was carried out for patients with middle and distal ureteral tumors, and a better LRFC was observed (χ2 = 5.248, P = 0.022). In most studies, tumor stage, grade or other variables, including age, tumor location, lymph node involvement, multifocality, and tumor architecture, were considered the most important prognostic factors for UTUC [32–34]. In this study, univariate analysis showed that R0 resection (χ2 = 4.098, P = 0.043), early stage (I + II) (χ2 = 6.485, P = 0.011) and low-risk population (χ2 = 4.079, P = 0.043) were associated with good OS, but multivariate analysis showed that age was the only factor of OS, perhaps due to the percentage of patients aged ≥ 75 years accounting for 22.2% (8/36). Therefore, for patients with kidney protection requirements (especially older patients), postoperative adjuvant radiotherapy may be a feasible choice.

## Conclusion

Adjuvant radiotherapy is safe and tolerated, and LRFS was superior in middle and distal ureteral cancer than in proximal ureteral cancer. With the development of immune checkpoint inhibitors in ureteral cancer, whether OS can be improved in KSS patients by immune combination with radiotherapy needs to be further explored.

## Data Availability

The data that support the findings of this study are available from the corresponding author upon reasonable request.
